# State Adoption of 100% Smoke-Free Acute Non Federal Hospital Campus Policies

**DOI:** 10.3390/ijerph6112793

**Published:** 2009-11-10

**Authors:** Adam O. Goldstein, Julea Steiner, Anna McCullough, Kathryn D. Kramer, Melva Fager Okun

**Affiliations:** 1 University of North Carolina, Department of Family Medicine/Chapel Hill, NC 27599, USA; E-Mails: jsteiner@med.unc.edu (J.S.); annamc@unc.edu (A.M.); kdkramer@med.unc.edu (K.D.K.); 2 Prevention Partners of North Carolina/Chapel Hill, NC 27514, USA; E-Mail: melva@ncpreventionpartners.org

**Keywords:** tobacco, hospital, policy, policy diffusion, smoke-free, tobacco-free, hospital campus, secondhand smoke, smoking, cessation

## Abstract

To assess the number and percentage of acute care hospitals in the U.S. that have adopted smoke-free hospital campus (SFHC) policies, researchers conducted an assessment from January 2008 to May 2008 of available data on SFHC policy adoption in each state. Slightly more than one third (34.4%) of acute care, non-Federal hospitals had adopted such policies, with wide variation of policy adoption between states.

## 1. Introduction

To achieve broad public health objectives related to tobacco use among adults in the U.S., increased implementation of strategies that augment tobacco cessation and eliminate secondhand smoke exposure are needed [1,2]. When the Joint Commission on Accreditation of Healthcare Organizations (JCAHO) required all accredited U.S. hospitals to prohibit smoking within their buildings, increased rates of smoking cessation occurred among employees [3], and the positive impacts of indoor smoke-free policies in hospitals on employee quit rates are well documented [4,5]. Efforts to increase cessation rates among hospital employees and patients and to increase availability of and access to cessation services have coincided with an increasing number of hospitals adopting SFHC or tobacco-free hospital campus (TFHC) policies. SFHC policies prohibit the use of cigarettes, pipes or cigars inside any hospital buildings or structures and also in all outdoor areas on the hospital campus. Tobacco free policies include all smoked tobacco products in addition to smokeless tobacco. SFHC policies offer greater protection from secondhand smoke exposure to patients, employees, volunteers and visitors by eliminating exposure to smoking on hospital campuses. Tobacco free policies add the element of deterring smokers from switching to smokeless. As no uniform method currently exists for collecting and disseminating data on U.S. hospitals that have adopted SFHC policies, researchers conducted a small-scale assessment from January 2008 to May 2008 to ascertain, retrieve and verify SFHC policy adoption. This protocol initially included a review of all state hospital association web sites for any information within the state about adoption of SFHC policies. Researchers attempted contact with a specific staff person within the state hospital association to discuss any tracking or verification mechanism. A second step involved contacting at least one tobacco control organization in each state to determine if any other organization outside of the state hospital association might track SFHC policy adoption in that state. Contacts in the state hospital or tobacco control organization confirmed whether data collection on SFHC policy adoption occurred within the state, who collected such data, which hospitals had adopted such policies or whether no systematic source existed for the collection of such data.

In cases where no information existed, researchers conducted systematic Internet searches using search terms of “tobacco free hospital campus” and “smoke free hospital campus”. Researchers confirmed the number of SFHC policy adoptions in these states by reviewing documentation generated from these searches, examining hospital web sites or by direct contact with hospital staff. Researchers also utilized a similar Internet verification procedure for states that had reports of five or less 100% SFHC policy adoption (n = 12).

Information about the total number of hospitals in the state came from data provided by the American Hospital Association, with updates by states, as needed, if more reliable information was available. For the purposes of this survey, only hospitals classified as acute care or community hospitals were included, and hospitals classified as psychiatric, long-term care and Federal were excluded.

Fifteen state hospital associations indicated they had data on the number of SFHCs in their state. Information for an additional twenty states came primarily through a source other than the hospital association (e.g., tobacco control group). For sixteen states, information on the status of SFHCs was not available from any state organization.

## 2. Results and Discussion

Data show that a little more than one third (34.4%) of U.S. acute care hospitals have adopted 100% SFHC policies. The data also indicate wide variation in the number of SFHC policy adoptions by state (Table 1). In twenty seven states, less than 25% of hospitals have adopted 100% SFHC policies. In only seven states (Arkansas, Wisconsin, Indiana, Ohio, Michigan, North Carolina and Iowa) have 75% or more of acute care hospitals adopted 100% SFHC policies.

Research on adoption of 100% smoke-free campus wide policies in schools and workplaces has shown that, if enforced, these policies decrease tobacco consumption [7,8]. While research documenting the successes or effectiveness of SFHC or TFHC policies on employees, patients, or visitors to U.S. hospitals is quite limited, the potential positive health effects are substantial [9]. This data systematically shows the extent of SFHC policy adoption in U.S. acute care hospitals.

Limitation to this data exist, including the fact that no uniform source exists for the retrieval of information documenting the existence of 100% SFHC or TFHC policies in states. A recent survey of hospitals seeking such data estimated a slightly higher number of hospitals in the U.S. as having SFHC policies (45% compared to 34% reported here), but that estimate was based on a sample response rate of only 43% and total reports of slightly less than one half the number of SFHC policies reported in our study. Further, no data was available for several states with too few responses [10]. To overcome these problems, we obtained data from multiple sources and relied on documentation from at least one source, focusing on records of policy adoption. Alternatively, our study did not collect secondary data on hospital characteristics, a factor that does help explain reasons associated with policy adoption [10]. Since our data were collected from organizations in addition to individual hospitals, it is likely that our data underestimate the number of hospitals that have adopted 100% SFHC or TFHC policies. For instance we discovered hospitals that had passed 100% SFHC polices, even when a state contact had indicated no such policies existed. On the other hand, overestimation of the extent of policy adoption could also occur when using self-report data alone, including having exceptions to such policies [10]. A strength of our methods is that they allow for repeated, rapid and efficient examination of SFHC policy adoption over time. Information regarding policy compliance for all of these policy adoptions in states is important but not currently available. A final limitation is that we limited data collection to hospitals designated as acute care; thus this data cannot verify the number of federal or psychiatric hospitals that have adopted 100% SFHC or TFHC policies.

Though an effective strategy, few states have pushed for or achieved universal 100% SFHC policies to date. Despite limited state activity, the SFHC movement is spreading across the U.S. with the rapid adoption of SFHC policies in several states. A similar process has occurred in Canada [11]. In most states, the process of policy adoption is occurring through grassroots activism and local leadership. For instance, in Michigan, the SFHC effort began with a statewide coalition encouraging SFHCs and increased smoking cessation programs in local communities and inpatient settings. Since 2000, the Michigan Department of Community Health has provided an annual grant to help increase smoke-free hospital campuses. In Wisconsin, the state hospital association encouraged all hospitals in 2006 to adopt 100% SFHC policies. In Arkansas a statewide law (passed in October, 2005) required all Arkansas hospital campuses, except psychiatric hospitals, to implement 100% SFHC policies, not including smokeless tobacco products [12].

In North Carolina, one organization has collected data on the passage of TFHC policies in the state for the last three years, allowing examination of the annual rate of TFHC policy adoption in one state [6]. North Carolina hospitals chose to develop and implement campus policies that address the use of all tobacco products, not just cigarettes.

**Figure 1.**
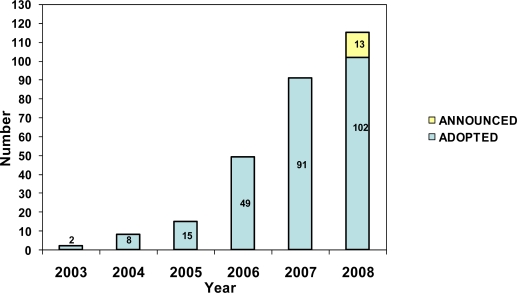
Cumulative adoption of TFHC policies in North Carolina acute care hospitals (Jan 2003–May 2008).

Data from North Carolina show rapid passage of 100% TFHC policies statewide in a short period of time. In 2006, the North Carolina Hospital Association (NCHA) and NC Prevention Partners (NCPP), with support from The Duke Endowment, created the Healthy Hospital Initiative to assist all North Carolina hospitals in adopting 100% TFHC policies and also to provide comprehensive cessation support to hospitals [6]. Of the state’s acute care hospitals, 102 passed TFHC policies from 2005 to 2008 (Figure 1). As of May 2008, another 13 had announced policies scheduled for implementation in 2009.

In North Carolina, prior to the 2006 inception of the Healthy Hospital Initiative, only 11% of North Carolina hospitals had adopted 100% SFHC policies. The goal of the Healthy Hospital Initiative was to have 100% of North Carolina’s hospitals adopt 100% TFHC policies by June 2009, a goal that was recently achieved. Strategies utilized by the Initiative include use of statewide opinion leaders, technical assistance, public education, model policies, peer networks of hospital administrators and personnel, and frequent dissemination of policy successes. Dissemination activities include a website (http://www.healthyhospital.org/) where information about the rate of policy adoption statewide is posted. Hospitals in other states can follow North Carolina in adopting “tobacco-free” versus “smoke-free” campus wide policies to assure that employees do not switch from using cigarettes or other smoke products to smokeless tobacco products. Benefits of these policies are immediate (decreased exposure to secondhand smoke for patients, visitors and staff) and sustained (reductions in employee and staff tobacco use) [13].

To facilitate the sharing of policy successes across states, the JCAHO recently launched WikiHealthCare (http://wikihealthcare.jointcommission.org), a collaborative forum for health care professionals to discuss issues including smoking cessation and SFHC policies. Resources provided by the JCAHO Wiki and the NC Healthy Hospital Initiative can foster the successful replication of state models of policy diffusion in hospitals and health care systems nationwide.

## 3. Conclusions

This data, combined with another recent study, shows that the spread of 100% SFHC policies across the U.S. is rapidly occurring [10]. In some states, such as Rhode Island, leaders have announced that all acute care hospitals in the state will implement 100% SFHC policies by the end of 2009. The adoption of SFHC and TFHC policies will better link new tobacco dependence treatment strategies with health care system interventions [14].

## Figures and Tables

**Table 1.** Percent of acute-care 100% smoke free hospital campuses by state, as of May, 2008.

State	Total	SFHC	%
75% or greater			
Arkansas	85	85	100.0%
Wisconsin	124	124	100.0%
Indiana	127	120	94.5%
Ohio	170	149	87.6%
Michigan	146	124	84.9%
North Carolina	122	102	83.6%
Iowa	116	94	81.0%

50%–74%			
Kansas	131	94	71.8%
Maryland	50	34	68.0%
Delaware	6	4	66.7%
Vermont	14	9	64.3%
New York	203	117	57.6%
Hawaii	25	14	56.0%
Alaska	22	12	54.5%

25%–49%			
Virginia	87	37	42.5%
Massachusetts	79	30	38.0%
Oklahoma	131	49	37.4%
Pennsylvania	182	67	36.8%
Oregon	58	19	32.8%
Maine	37	11	29.7%
Nebraska	87	25	28.7%
Mississippi	94	27	28.7%
Rhode Island	11	3	27.3%
Colorado	71	19	26.8%

Less than 24%			
West Virginia	57	14	24.6%
Arizona	67	16	23.9%
Kentucky	105	25	23.8%
Montana	54	12	22.2%
Idaho	39	8	20.5%
Tennessee	135	27	20.0%
Georgia	172	32	18.6%
District of Columbia	11	2	18.2%
Wyoming	24	4	16.7%
Missouri	119	19	16.0%
South Carolina	63	9	14.3%
New Hampshire	28	4	14.3%
California	357	50	14.0%
New Jersey	80	11	13.8%
Minnesota	133	17	12.8%
Illinois	191	24	12.6%
Florida	229	28	12.2%
Alabama	109	11	10.1%
Louisiana	128	11	8.6%
Nevada	49	3	6.1%
South Dakota	52	3	5.8%
Connecticut	36	2	5.6%
New Mexico	37	2	5.4%
Texas	415	22	5.3%
Washington	86	4	4.7%
North Dakota	40	1	2.5%
Utah	43	1	2.3%

Total	5037	1731	34.4%
